# Unraveling the Antioxidant Capacity of *Spatholobi caulis* in Nonalcoholic Fatty Liver Disease: A Multiscale Network Approach Integrated with Experimental Validation

**DOI:** 10.3390/antiox12051097

**Published:** 2023-05-13

**Authors:** Su-Jin Bae, Won-Yung Lee, Seon-Been Bak, Young-Eun Kim, Min-Jin Kim, Young-Woo Kim

**Affiliations:** 1 School of Korean Medicine, Dongguk University, Gyeonju 38066, Republic of Korea; realsujin@dgu.ac.kr (S.-J.B.); wonyung21@dongguk.ac.kr (W.-Y.L.); sbpark@dongguk.ac.kr (S.-B.B.); yekim@dgu.ac.kr (Y.-E.K.); mjkim@dgu.ac.kr (M.-J.K.); 2 Department of Computer Science and Engineering, Kyungpook National University, Daegu 41566, Republic of Korea

**Keywords:** oxidative stress, non-alcoholic fatty liver diseases, *Spatholobi caulis*, multiscale network

## Abstract

Nonalcoholic fatty liver disease (NAFLD) is a global health problem that is closely associated with obesity and metabolic syndrome. *Spatholobi caulis* (SC) is a herbal medicine with potential hepatoprotective effects; however, its active compounds and underlying mechanisms have not been fully explored. In this study, we combined a multiscale network-level approach with experimental validation to investigate SC’s antioxidant properties and their impact on NAFLD. Data collection and network construction were performed, and active compounds and key mechanisms were identified through multi-scale network analysis. Validation was conducted using in vitro steatotic hepatocyte models and in vivo high-fat diet-induced NAFLD models. Our findings revealed that SC treatment improved NAFLD by modulating multiple proteins and signaling pathways, including AMPK signaling pathways. Subsequent experiments showed that SC treatment reduced lipid accumulation and oxidative stress. We also validated SC’s effects on AMPK and its crosstalk pathways, emphasizing their role in hepatoprotection. We predicted procyanidin B2 to be an active compound of SC and validated it using a lipogenesis in vitro model. Histological and biochemical analyses confirmed that SC ameliorated liver steatosis and inflammation in mice. This study presents SC’s potential use in NAFLD treatment and introduces a novel approach for identifying and validating active compounds in herbal medicine.

## 1. Introduction

Non-alcoholic fatty liver disease (NAFLD) refers to a condition marked by the accumulation of fat (mainly over 5%) in the liver tissue of patients who do not consume large quantities of alcohol. NAFLD is a typical liver metabolic disorder and spans various conditions, such as simple steatosis, steatohepatitis, liver fibrosis, and hepatocellular carcinoma [1]. Recent research indicates that 25% of the general population has NAFLD, with higher occurrence rates in high-income countries [2]. Although the precise pathophysiological mechanism of NAFLD is not fully understood, the most widely accepted concept is the “multiple-hits model”, involving concurrent factors such as fat accumulation, inflammatory reactions, and oxidative stress [3]. Specifically, oxidative stress plays a crucial role in the pathogenesis and progression of NAFLD, as it can cause lipid peroxidation, cellular damage, and the release of pro-inflammatory cytokines [4]. NADPH oxidase (NOX), which purposefully produces ROS, has been reported to lead to the pathogenesis of NAFLD by causing mitochondrial oxidative phosphorylation dysfunction and lipid accumulation in liver cells [5]. NOX is also involved in the activation of hepatic stellate cells that cause liver fibrosis, and this process itself induces the upregulation of various NOX isoforms (NOX1, NOX2, NOX4), creating a vicious circle and potentially leading to the progression to hepatocellular carcinoma [6,7]. While NAFLD has become one of the most pressing medical issues worldwide, there is no effective therapeutic approach. Therefore, targeting oxidative stress and its associated signaling pathways may provide a promising therapeutic approach for the treatment of NAFLD.

*Spatholobi caulis* (SC), which is the dried vine of *Spatholobus suberectus* Dunn, belonging to the Leguminosae family, has been used as a traditional herbal medicine [8]. SC has been reported to exhibit effects on blocking tumor cell aggregation and migration, promoting blood circulation, and also showing potential in preventing deep vein thrombosis through its anti-inflammatory effects via the upregulation of SIRT1 and nuclear factor erythroid 2–related factor 2 (Nrf2) expression [9,10,11]. In particular, SC treatment was found to upregulate MAPK and AMPK pathways in white adipose tissue and brown adipose tissue, resulting in anti-obesity effects in high-fat diets (HFD)-induced in obese mice [12]. Additionally, SC exhibited the potential to protect liver cells from oxidative stress-induced damage. In our recent research, we reported that SC alleviated acute liver injury induced by drug overdose in vivo and protected liver cells against acute oxidative stress-induced damage through both AMPK and Yes-associated protein (YAP) pathways in vitro [13]. The accumulation of body fat increases adipokines, which leads to the overproduction of reactive oxygen species (ROS) and, eventually, to oxidative stress [14]. Oxidative stress is known to contribute to the development and progression of many chronic diseases, including NAFLD. However, there is limited research on the effects and mechanisms of SC on liver damage induced by oxidative stress in NAFLD.

Network pharmacology, inspired by a systems biology approach, has emerged as a potent tool for recognizing rational drug targets and repurposing drugs [15]. This approach offers notable benefits in unraveling disease treatment mechanisms within interconnected biological networks, enabling researchers to overcome the constraints of the single-target paradigm [16]. Network pharmacology is especially valuable for investigating the complex mechanisms of herbal medicines, which often exhibit multi-compound, multi-targeting properties. Indeed, researchers have utilized network pharmacology to identify the inhibitory effects of cordycepin on breast cancer, the mode of action for the guizhi-fuling capsule on primary dysmenorrhea, and the antioxidant capacities of Bupleuri Radix [17,18,19]. Network-based approaches can also be utilized to explore novel indications for drugs and their key mechanisms. A recent study highlighted that disease treatment mechanisms could be more accurately identified by considering their biological functions through which target proteins can modulate the functions of disease-related proteins [20]. This research indicated that drug-disease associations and their key mechanisms could be more precisely identified by taking into account the impact of drugs and diseases at the multiscale network level. These findings suggest that network pharmacology could present a novel opportunity for uncovering the key mechanisms and active compounds in herbal medicine for disease treatment.

In this study, we investigated the antioxidant properties of SC and its impact on NAFLD by combining a multiscale network-level approach with experimental validation. An integrated approach that combines computational and experimental methods was employed to evaluate the hepatoprotective effects of SC, as well as the key mechanisms and active ingredients underlying them. To this end, we first collected information on the detectable ingredients of SC and their experimentally validated targets. By analyzing the collected dataset, we identified key targets of SC and signaling pathways that were significantly associated with these key targets. The signaling pathways associated with this proposed therapeutic effect were comprehensively validated using an in vitro oxidative stress model. Furthermore, in vivo experiments confirmed that the hepatoprotective effect of SC could be achieved through oral administration. To identify the active ingredients, we utilized protein overlap and multiscale-level approaches to identify the association between SC ingredients and the disease. The mechanisms of the candidates were proposed by analyzing diffusion profiles and were further validated by subsequent experiments. Through this approach, we sought to contribute to the development of more effective therapeutic strategies for NAFLD and provided valuable insights into the potential applications of herbal medicines.

## 2. Materials and Methods

### 2.1. Compound-Target Network Construction

A compound-target network was constructed by identifying compounds and their protein targets. These compounds were retrieved from the Oriental Advanced Searching Integrated System (OASIS, https://oasis.kiom.re.kr. accessed date: 26 February 2023) database [21]. OASIS is a database of Korean medicine that provides information on traditional medicine studies and the active compounds of herbal medicine. Of the components of the SCs included in the databases, we considered only compounds that could be mapped to the PubChem ID. Protein targets were obtained from assembled datasets of experimentally validated compound–target interactions. The experimentally validated targets were obtained from the Drugbank, therapeutic target database (TTD, version 1.0), Search Tool for ITteractions of Chemicals (STITCH, version 5.0), and a database assembled by Huang et al. [22,23,24,25]. DrugBank and TTD provided comprehensive information about the known and explored targets, the targeted disease, pathway information, and the corresponding drugs directed at each of these targets. STITCH integrated target information for 430,000 chemicals from disparate data sources. Huang et al. assembled direct and indirect compound–protein interactions (CPIs) of natural products from several databases. After gathering target information for the ingredient of the SCs in each database, target identifiers such as gene symbols or UniProt IDs were mapped and unified with the Entrez gene IDs using SYNGO [26].

### 2.2. Disease-Associated Protein and Signaling Pathway

Proteins associated with liver injury and oxidative stress were obtained using STRING PubMed query in the Cytoscape StringApp [27]. This plugin, based on text-mining technology, displays a set of proteins that are related to user-defined queries. It is useful for identifying disease-associated proteins by concentrating on specific mechanisms, as demonstrated by a previous study that explored the systems-level mechanisms of *Bupleuri Radix* on oxidative stress [28]. The search string ‘liver injury’ AND ‘oxidative stress’ was employed. Genes/proteins with co-occurrence scores above the threshold were chosen as liver injury-related proteins linked to oxidative stress. To select reliably related proteins, the threshold was set to 1, which is higher compared to previous studies [28]. The chosen proteins were imported as a STRING network into Cytoscape 3.7 for subsequent network analysis.

Liver injury-related signaling pathways identified in the literature included the following [29,30,31]: AMPK, JAK-STAT, MAPK, NF-κB, PI3K-Akt, TNF, Toll-like receptor, apoptosis, and protein processing in the endoplasmic reticulum. Pathways associated with herbal targets were determined using enrichment analysis provided by Enrichr [32]. Enrichr computes enrichment by evaluating various gene-set libraries, including gene ontology (GO), Kyoto Encyclopedia of Genes and Genomes (KEGG), and Online Mendelian Inheritance in Man (OMIM). It then calculates the adjusted *p*-values and combined scores for the gene list of interest, which consists of the target genes. The combined score is determined as the logarithm of the product of the *p*-value and z-score. The Benjamini-Hochberg procedure was employed to correct the false discovery rate arising from multiple tests.

### 2.3. Multiscale Network-Level Analysis

The multiscale network employed in this study was derived from Ruiz et al. [20]. The network was constructed by integrating three types of associations: protein–protein, protein–biological function, and biological–biological function interactions. To achieve this, various interaction information was collected and assembled from major databases such as the Biological General Repository for Interaction Datasets, the Database of Interacting Proteins, and Human Reference Protein Interactome Mapping Project Gene Ontology. The network comprised 387,626 physical interactions among 17,660 proteins, 34,777 associations between 7993 proteins and 6387 biological functions, and 22,545 associations between 9798 biological functions.

The active compounds of the disease were identified by calculating and analyzing diffusion profiles, which encoded the drug or disease effects on proteins and biological functions. A diffusion profile was computed through a matrix formulation with power iteration. The process involved variables such as the probability that a random walker continued to walk at a given step rather than restarting and a restart vector indicating the probability that the walker would jump to each node after a restart. Furthermore, the process utilized a directed multiscale network-derived biased transition matrix with a set of scalar weights that encoded the relative likelihood of visiting other nodes. These procedures were repeated until the convergence of the power iteration computation met a specific tolerance parameter. Ultimately, Pearson’s correlation of drug and disease diffusion profiles was calculated, considering the diffusion profiles of the drug and the disease.

The key mechanisms for a given compound-disease pair were identified by analyzing their diffusion profiles. The top k-proteins or biological functions in the compound and disease diffusion profiles were selected as those most affected by each drug or disease. A subnetwork consisting of the selected nodes was constructed to elucidate the relevance of these proteins and their biological functions. Compound targets not connected to disease-associated proteins or biological functions, or vice versa, were excluded. The highest-ranking node in the diffusion profile was considered the most relevant for treatment, as this was the most affected by the component or disease. The value of k was set at 20 to capture a significant fraction of the overall visit frequency in the diffusion profile. Detailed information regarding the active ingredients and key mechanisms could be found in previous research [20].

### 2.4. Animals and Treatment

The animal experimentation procedures of this study were approved and monitored by the Institutional Animal Care and Use Committee of Dongguk University. Male C57BL/6 mice (6 weeks, 18–20 g) obtained from Charles River Orient Bio (Seongnam, Republic of Korea) were randomly divided into 3 groups: Normal diet (ND) + vehicle, HFD + vehicle, HFD + SC 30 mg/kg. Mice were fed either ND (PicoLab^®^ Rodent Diet 20 5053) or HFD (comprising 60% calories from fat, using product D12492 from the Research Diet), for 4 weeks, depending on the group. Subsequently, mice were orally injected with a vehicle (40% PEG) or SC (30 mg/kg, dissolved in 40% PEG) three times a week for 5 weeks. All mice were fasted from the last administration until sacrifice.

### 2.5. Blood Chemistry

Aspartate aminotransferase (AST), triglyceride, and LDL cholesterol levels were analyzed at Chaon Research Lab (Seongnam, Republic of Korea) using Diagnostics Reagents for AST and triglyceride (Chema-Diagnostica, Monsano, AN, Italy) and Diagnostics Reagents for LDL cholesterol (FUJIFILM Wako Pure Chemical Corporation, Osaka, Japan) on an AU680 chemistry analyzer (Beckman Coulter, CA, USA).

### 2.6. Hematoxylin and Eosin and Oil Red O Staining

For haematoxylin and eosin (H&E) staining, liver tissue samples isolated from the left lateral lobe were fixed in 4% paraformaldehyde in PBS at room temperature for 24 h. The fixed liver tissue samples were then dehydrated in a sequential gradient of alcohol and xylene before it was embedded in paraffin. Subsequently, the paraffin-embedded tissues were sectioned into 5 μm thick slices. Hematoxylin and eosin (H&E) staining was performed on the sectioned liver tissues. For Oil Red O staining, the left lateral lobe of the liver was frozen-sectioned and fixed in 4% paraformaldehyde for 30 min. After washing with tap water, the tissue was rinsed in 60% isopropanol and then stained with fresh Oil Red O solution for 10 min. The tissue was then rinsed in 60% isopropanol again, followed by the counterstaining of cell nuclei with hematoxylin. Finally, the stained tissues were observed using light microscopy (Nikon, Tokyo, Japan).

### 2.7. Chemicals and Reagents

Anti-SREBP-1c, anti-phospho-AMPKα, anti-phospho-acetyl-CoA carboxylase (ACC), anti-phospho- liver kinase B1 (LKB1), anti-phospho-YAP, anti-YAP, anti-phospho-large tumor suppressor kinase 1 (LATS1), anti-heme oxygenase 1 (HO-1), anti-Lamin A/C, and anti-β-actin were purchased from Cell Signaling Technology (Danvers, MA, USA). Anti-Nrf2 was purchased from Santa Cruz Biotechnology (Dallas, TX, USA). HRP-conjugated anti-rabbit IgG and HRP-conjugated anti-mouse IgG were purchased from Enzo Life Sciences (Farmingdale, NY, USA). Trizol reagent was purchased from Invitrogen (Carlsbad, CA, USA). Compound C (Com C), verteporfin (VP), T0901317 (T090), Harris hematoxylin and eosin, and Oil Red O solution were purchased from Sigma-Aldrich (St. Louis, MO, USA). Polyethylene glycol (PEG) 400 was obtained from Yakury Pure Chemical Co., Ltd. (Kyoto, Japan). SC is a medicinal-standard herb manufactured at a GMP facility (Nonglim Saengyak, Seoul, Republic of Korea) certified by the Korean FDA and was prepared and standardized as previously described [13].

### 2.8. Cell Lines and Cell Culture

HepG2 and Huh-7 cells (American Type Culture Collection, Rockville, MD, USA) were cultured in Dulbecco’s modified Eagle’s medium (DMEM) at high glucose with 10% fetal bovine serum (FBS), 50 μg/mL streptomycin, and 50 units/mL penicillin and RPMI 1640 with 10% FBS, 50 μg/mL streptomycin, and 50 units/mL penicillin, respectively.

### 2.9. Quantitative Real-Time PCR

The total RNA was extracted from liver tissue using the Trizol reagent. cDNA was reverse transcribed using the cDNA Synthesis Kit (NanoHelix Co., Ltd., Daejeon, Republic of Korea). The reaction mixture, including cDNA, forward and reverse primers, and the Premier qPCR Kit (NanoHelix Co., Ltd., Daejeon, Republic of Korea), was amplified using Light Cycler 1.5 (Roche, Mannheim, Germany). The sequences of primers were then as follows: mouse NOX-4(Entrez Gene ID: 50490)-F: 5′-GATCACAGAAGGTCCCTAGCA-3′, NOX-4-R: 5′-GTTGAGGGCATTCACCAAGT-3′, GAPDH (Entrez Gene ID: 14433)-F: 5′-AACTTTGGCATTGTGGAAGG-3′, GAPDH-R: 5′-ACACATTGGGGGTAGGAACA-3′.

### 2.10. Immunoblot Analysis

HepG2 cells were seeded in a 60Ø dish at a density of 1.6 × 10^6^ and then treated with SC at the concentrations and times indicated in each figure. For cell lysate preparation, the RIPA buffer was used at 4 °C, and the protein quantification of lysates was performed using a BCA assay kit (Thermo Fisher Scientific Inc., Waltham, MA, USA). The quantified proteins were separated by sodium dodecyl sulfate-polyacrylamide gel electrophoresis (SDS-PAGE) and transferred to a PVDF membrane for subsequent antibody conjugate probing. The membrane was then incubated with primary and secondary antibodies, and chemiluminescence was detected using a Chemidoc image analyzer (Vilber Lourmat, Marne La Vallée, France) to visualize the protein-bound membrane.

### 2.11. Statistical Analysis

Statistical analysis was conducted using Student’s t-test or ANOVA to determine significant differences between the control and treatment groups. Blood chemistry data from animal experiments, including AST, triglyceride, and LDL cholesterol values, were presented as the mean ± standard deviation (SD). The criterion for statistical significance was set at *p* < 0.05 or *p* < 0.01.

## 3. Results

### 3.1. Construction and Analysis of Compound-Target Network

We first performed a network pharmacological analysis to investigate the complex relationships between the compounds, targets, and biological effects of SC on NAFLD. This initial analysis provided a comprehensive overview of the possible interactions between SC ingredients and their protein targets in the context of NAFLD, setting the stage for further investigations into the specific mechanisms involved. A compound-target network of SC was constructed by collecting ingredients and their target information from various databases. We initially found 589 CPIs between 21 SC ingredients and 321 protein targets and 91 liver injury-related proteins associated with oxidative stress. Among the protein targets of SC, we found that CYP19A1, NFKB1, CASP3, NOS2, CA12, ESR2, and PTGS2 had the highest degree (9, 8, 8, 7, 7, 7, and 6, respectively). On the other hand, we found that around two-thirds of the protein targets (210/321) interacted with only one SC target. To focus on the protein targets associated with a sufficient number of SC ingredients, we considered 111 targets that interacted with two or more ingredients as the key targets and conducted subsequent analyses focusing on them.

We then investigated the association between key targets in SC and NAFLD and oxidative stress-related proteins and found that approximately 20% of the proteins (27/111) overlapped with oxidative liver injury-associated targets. A hypergeometric test was conducted to check whether the observed number of overlapping targets was higher than random expectations. The values of random expectation were obtained by randomly selecting targets that were equal to the number of key targets from the assembled dataset and then repeatedly calculating the number of overlapping targets between the disease-related proteins and the selected targets. We found that the observed value was significantly higher (22-fold) than the random expectations (*p*-value < 10^−30^). This result indicated that the key targets of SC were closely associated with oxidative stress, highlighting the potential of SC as a therapeutic agent for NAFLD.

We then investigated which relevant signaling pathways the SC targets were associated with. We performed an over-representation analysis based on the KEGG database and found that all signaling pathways were significantly associated with the main targets of SC, except for one of the pathways that were known to be involved in oxidative damage and NAFLD (Table 1). In particular, we found that the AMPK pathway, which may have contributed to the treatment of NAFLD by inducing apoptosis, was also closely associated with the key targets of SC.

**Figure 1:**
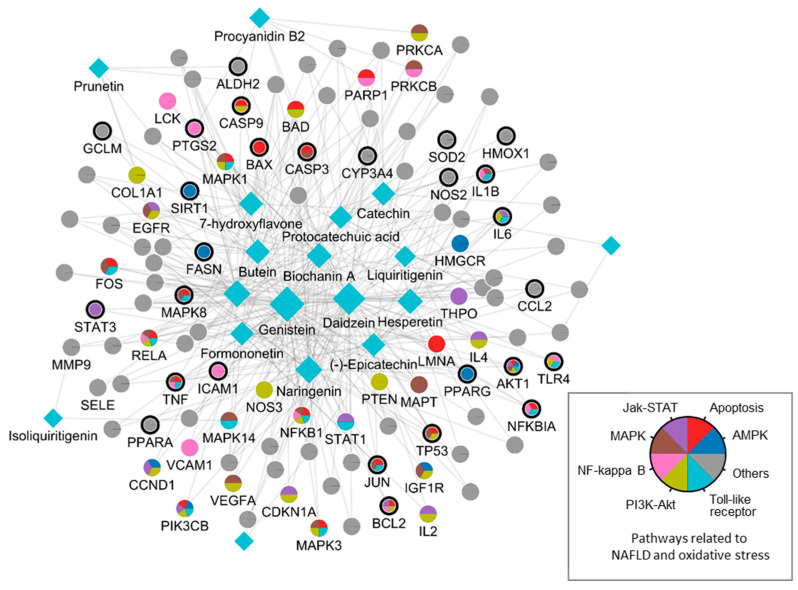
A compound-target network of SC. Circles and diamonds denote protein targets and compounds, respectively. The circle edge and body color indicate targets and signaling pathways relate to oxidative stress and NAFLD, respectively. Edges denote interactions between the compound and target.

By integrating information about the identified ingredients, key protein targets, and the associated pathways of SC, we constructed and visualized the compound-target network (Figure 1). The network consisted of 379 CPIs between 111 proteins and 20 SC ingredients. In the network, nodes represented RG ingredients and their targets, whereas edges represented the interactions between SC ingredients and protein targets. The number of related targets for the PI3K-Akt signaling pathway, MAPK signaling pathway, Apoptosis, Toll-like receptor signaling pathway, NF-kappa B signaling pathway, JAK-STAT signaling pathway, and AMPK signaling pathway were 23, 19, 19, 16, 13, 12, and 8, respectively. Specifically, we found that BCL2, TNF, NFKB1A, TLR4, IL1B, IL6, TP53, MAPK8, and CASP9 were simultaneously involved in NAFLD with oxidative stress and its related signaling pathways. These key proteins, as well as those involved in multiple signaling pathways, highlighted the complex and multifaceted nature of the therapeutic potential of SC in the treatment of oxidative stress-associated NAFLD. To further validate these findings, we subsequently sought to investigate the therapeutic effects and the underlying mechanisms of SC in NAFLD treatment using in vitro and in vivo experiments.

### 3.2. SC Alleviated High-Fat Diet-Induced Fat Accumulation In Vivo and Suppressed SREBP-1c Expression In Vitro

**Figure 2:**
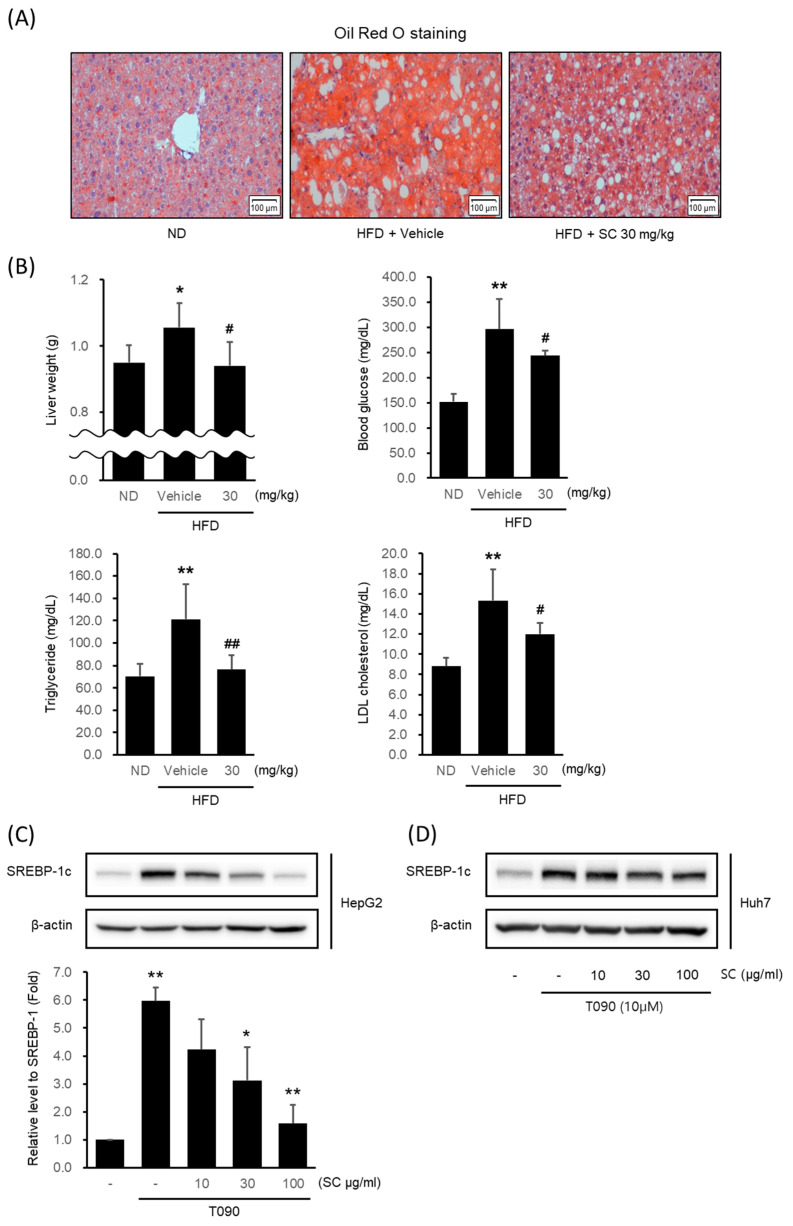
Effect of SC on high fat diet-induced fat accumulation in vivo and SREBP-1c expression in vitro. (**A**) Representative mouse liver images from each group with Oil Red O staining (scale bar represents 100 μm). Mice were fed a ND or a HFD for 9 weeks according to their respective groups. SC was administered orally at a dose of 30 mg/kg three times per week for 4 weeks, along with each respective diet. (**B**) Liver weight, blood markers of diabetes, including fasting blood glucose, and blood markers of hyperlipidemia, including triglycerides and LDL-cholesterol. The differences in Liver weight, fasting blood glucose, triglycerides, and LDL cholesterol were represented as statistical significance compared to the ND-fed group (* *p* < 0.05, ** *p* < 0.01) and the HFD-fed group (# *p* < 0.05, ## *p* < 0.01). Immunoblotting analysis of HepG2 cell (**C**) and Huh-7 cell (**D**) lysates for SREBP-1c protein. HepG2 and Huh7 cells were pre-treated with SC at concentrations of 10, 30, and 100 μg/mL for 1 h prior to incubation with T090 (10 μM) for 12 h.

To verify the potential of SC as a therapeutic agent for NAFLD, we confirmed its effectiveness in inhibiting lipogenesis in a high-fat diet-induced obesity model. Mice fed an HFD for 4 weeks were given 30 mg/kg SC orally for 5 weeks in combination with an HFD. The livers of HFD-fed mice showed fat accumulation via Oil Red O staining (Figure 2A). However, 30 mg/kg SC treatment reduced fat accumulation. The liver weight, fasting blood glucose level, blood triglyceride level (TG), and LDL cholesterol level elevated by HFD were also suppressed by 30 mg/kg SC treatment (Figure 2B). However, the body weight and periepididymal adipose tissue weight increased by HFD were not significantly decreased by 30 mg/kg SC treatment.

Next, we determined whether SC inhibited lipogenesis in the hepatocytes in vitro model. The expression of SREBP-1c, a specific transcription factor regulating lipogenesis, was significantly increased in HepG2 cells (Figure 2C) and Huh7 cells (Figure 2D) by T090: a synthetic agonist of LXRα. Pretreatment with 10–100 μg/mL SC decreased it in a concentration-dependent manner. Considering that the concentration of 100 μg/mL SC exhibited the most pronounced effects, this concentration was employed in subsequent experiments.

### 3.3. SC Activated the AMPK Signaling Pathway and the Hippo-YAP Signaling Pathway

**Figure 3:**
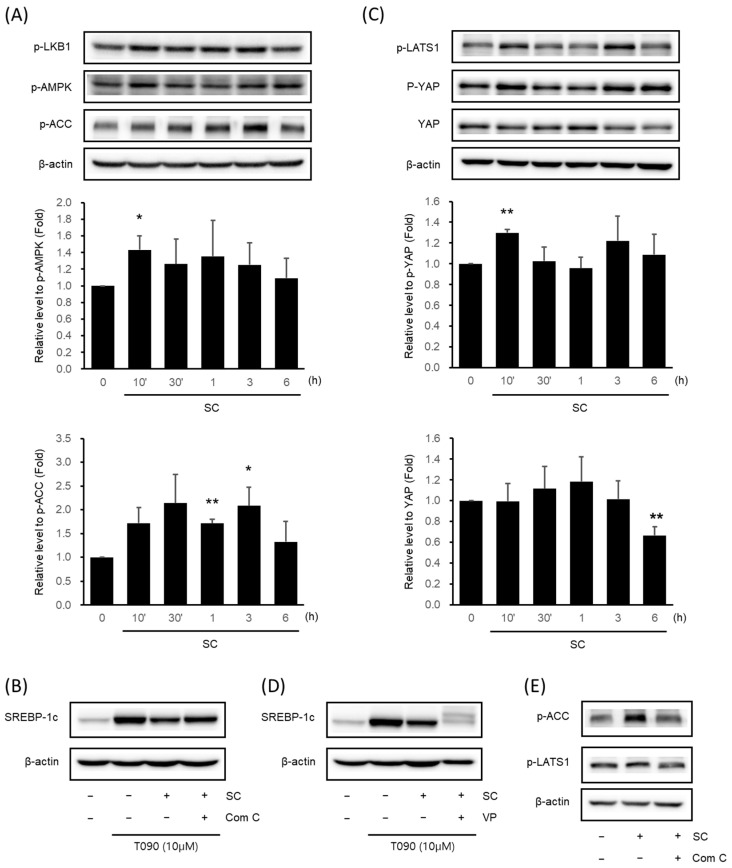
Effect of SC on activation of AMPK signaling pathway and Hippo-YAP signaling pathway. Immunoblotting analysis of key proteins in the AMPK pathway (**A**) and Hippo-YAP pathway (**C**). HepG2 cells were cultured in serum-free media for 12 h, followed by treatment with 100 μg/mL of SC for the indicated time period. Key proteins in each pathway, p-AMPK, p-ACC, p-YAP, and YAP, were tested in at least three replicates. Results were expressed as the mean ± SD, and statistical significance was indicated as * *p* < 0.05, ** *p* < 0.01 when comparing control and SC-treated cells. (**B**) Differences in SREBP-1c expression by Compound C: an AMPK inhibitor. HepG2 cells were treated with 10 μM Compound C for 30 min prior to treatment with 100 μg/mL SC for 1 h, followed by treatment with T090 (10 μM) for 12 h. (**D**) Changes in SREBP-1c expression by the YAP inhibitor, VP. HepG2 cells were treated with 0.6 μM VP for 30 min prior to treatment with 100 μg/mL SC for 1 h, followed by treatment with T090 (10 μM) for 12 h. (**E**) Expression of p-LATS1, a key protein in the Hippo-YAP pathway, by AMPK inhibition. HepG2 cells were treated with 10 μM Compound C for 30 min, followed by treatment with 100 μg/mL SC for 30 min.

We validated the key signaling pathways associated with oxidative stress, and NAFLD was predicted via network analysis by immunoblotting analysis in vitro. SC (100 μg/mL) increased the expression of p-LKB1, p-AMPK, and p-ACC. In particular, AMPK phosphorylation was significantly highest at 10 min, and ACC phosphorylation was significantly highest at 1–3 h (Figure 3A). To determine the influence of AMPK on the expression of SREBP-1c, we administered a pre-treatment of Compound C (Com C), an AMPK inhibitor, prior to treating the cells with SC. SC suppressed the upregulation of SREBP-1c expression induced by T090, but when administered after pre-treatment with Com C, it caused a reversal of the decrease in SREBP-1c expression (Figure 3B).

We further identified the Hippo-YAP signaling pathway, which is associated with the AMPK signaling pathway and was recently reported to be involved in lipogenesis. SC increased the expression of p-LATS1 and p-YAP and decreased the expression of YAP. In particular, p-YAP was significantly highest at 10 min, and YAP was significantly lowest at 6 h (Figure 3C). To investigate the influence of the Hippo-YAP signaling pathway on SREBP-1c expression, cells were pre-treated with the YAP inhibitor, VP, prior to treatment with SC. SC inhibited the T090-induced upregulation of SREBP-1c expression, and pre-treatment with VP prior to SC resulted in a further reduction in SREBP-1c expression (Figure 3D).

To investigate the influence of AMPK regulation on the Hippo-YAP signaling pathway, pre-treatment with Com C resulted in the decreased phosphorylation of ACC, a downstream kinase of AMPK, and the decreased phosphorylation of LATS1: an upstream kinase of YAP (Figure 3E).

### 3.4. Effects of SC on HFD-Induced Hepatic Injury In Vivo and Antioxidant Properties In Vitro

**Figure 4:**
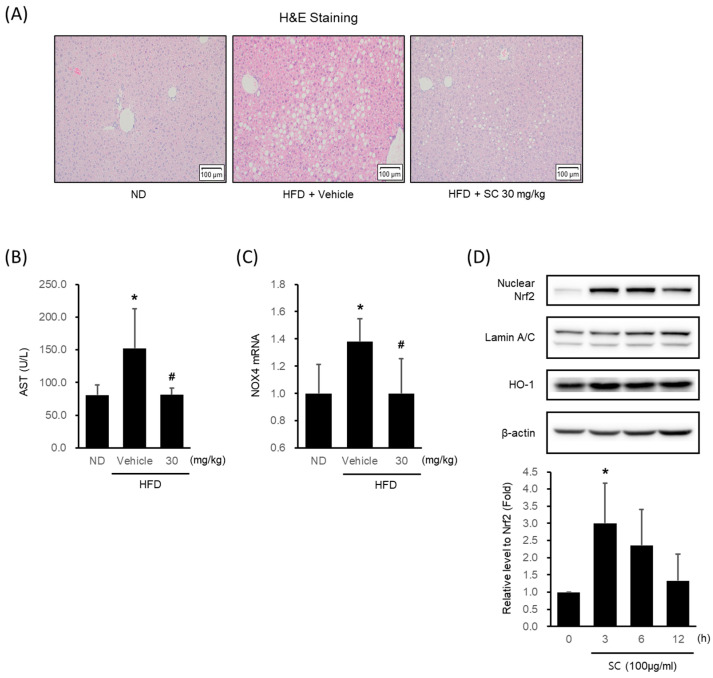
Effect of SC on high fat diet-induced hepatic injury in vivo and antioxidant-related proteins in vitro. (**A**) H&E images of liver tissue from a representative mouse in each group (scale bar represents 100 μm). Mice were fed as described in Figure 2A. (**B**) Blood markers of liver injury. The levels of aspartate aminotransferase were measured in the blood serum of mice from the respective group. (**C**) mRNA expression of NOX4 in liver tissue. The extracted mRNA from mouse livers in the respective groups was analyzed by quantitative real-time PCR. Statistical significance of aspartate aminotransferase levels in plasma and NOX4 mRNA levels in liver tissue was indicated by comparison with the ND-fed group (* *p* < 0.05) and HFD-fed group (# *p* < 0.05). (**D**) Immunoblotting analysis of Nrf2 in HepG2 cell nucleus. HepG2 cells were incubated in a serum-free media for 12 h, followed by treatment with 100 μg/mL of SC for 3, 6, and 12 h. Lamin A/C are loading controls of nucleus proteins for immunoblotting analysis. (**D**) Immunoblotting analysis of the expression HO-1 by SC. HepG2 cells proceeded as described in Figure 4D.

HFD-fed mice showed a marked formation of micro and macrovacuolation in hepatocytes, as observed in H&E staining, whereas 30 mg/kg SC treatment improved these phenomena (Figure 4A). HFD induced an elevation in AST levels, a blood-based marker of hepatic damage, while treatment with SC suppressed the increase in AST levels (Figure 4B). The HFD-induced increase in the expression of NOX4 mRNA in liver tissue was restrained by SC treatment (Figure 4C).

In HepG2 cells, SC treatment increased the expression of Nrf2, a key transcription factor of the antioxidant system that regulates the induction of antioxidant genes, with the highest expression observed at 3 h (Figure 4D). SC treatment also led to an increase in the expression of HO-1, an Nrf2-mediated phase II antioxidant enzyme (Figure 4D).

### 3.5. Identifying Active Ingredients of CS Using Multiscale Network-Level Analysis

We subsequently predicted the active compounds of SC by considering protein overlap and multiscale network analysis (Table 2). The protein overlap approach was based on the tendency of compounds with overlapping targets of disease-related proteins to exhibit therapeutic effects on the disease. A multiscale network encompasses a network of physical interactions between proteins and a hierarchy of biological functions. Analyzing multiscale interactors can capture the propagation effects of compounds or diseases on both proteins and biological functions. A more detailed procedure for calculating diffusion profiles on a multiscale network is described in the Methods section (Section 2). The overlap analysis suggested that eleven out of the fourteen ingredients exhibited significantly higher overlapping proteins compared to chance levels. This indicated that multiple ingredients in SC had the therapeutic potential for NAFLD and oxidative stress. The results of the multiscale network analysis also prioritized SC ingredients that were highly correlated with the diffusion profile of NAFLD and oxidative stress. These findings suggested that the prioritized ingredients may have exhibited a beneficial impact on the proteins and biological functions affected by NAFLD and oxidative damage.

**Figure 5:**
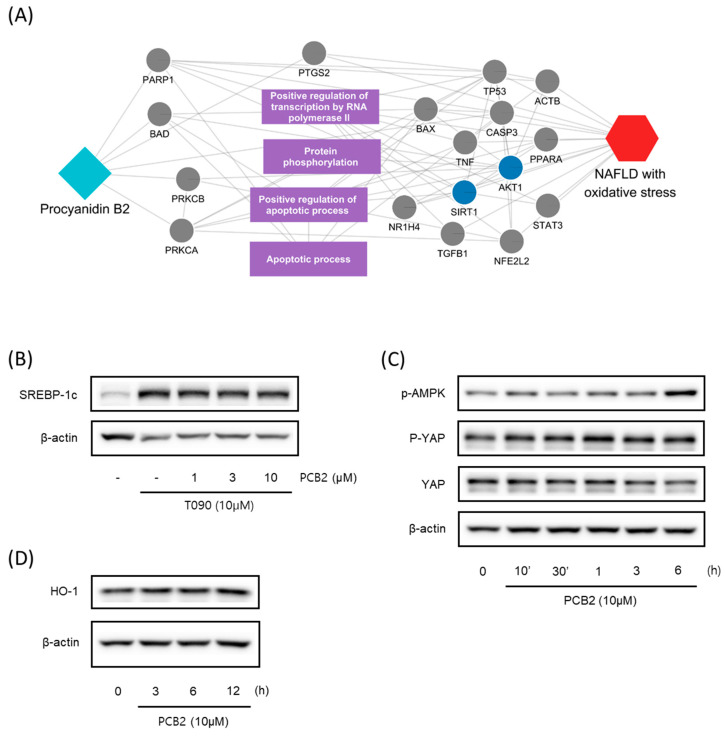
Multiscale network mechanisms and effects of Procyanidin B2 on AMPK and Hippo-YAP pathways. (**A**) Key mechanisms of procyanidin B2 on NAFLD with oxidative stress based on diffusion profiles in the multiscale network. Circle edges indicate targets related to liver injury. Blue colors indicate proteins related to the AMPK signaling pathways. (**B**) Immunoblotting analysis of HepG2 cell lysates for SREBP-1c protein. HepG2 cells were pre-treated with Procyanidin B2at concentrations of 1, 3, and 10 μM for 1 h and then incubated with T090 (10 μM) for 12 h. (**C**) Immunoblotting analysis of key proteins in the AMPK pathway and Hippo-YAP pathway. HepG2 cells were cultured in serum-free media for 12 h, followed by treatment with 10 μM of Procyanidin B2 for the indicated time period. (**D**) Immunoblotting analysis of the expression HO-1 by Procyanidin B2. HepG2 cells were treated with 10 μM Procyanidin B2 for 3, 6, and 12 h. PCB2; procyanidin B2.

We further investigated the hepatoprotective effects of SC ingredients. Among the prioritized active compounds, we focused on Procyanidin B2, as this is not only a potential quality marker for SC but was also easily detectable by chromatography technique [33,34]. We first investigated the multiscale-level mechanisms of procyanidin B2 against NAFLD with oxidative stress. We constructed a subgraph comprising the most frequently visited nodes in the diffusion profile of procyanidin B2 and NAFLD with oxidative stress (Figure 5A). The constructed network suggested that procyanidin B2 directly interacted with the protein PTGS2, which could be associated with NAFLD and liver oxidative damage. Additionally, we found that the protein targets of procyanidin B2 closely interacted with the biological functions related to transcription and apoptosis, as well as proteins related to the AMPK signaling pathway. These findings suggest the potential mechanisms of procyanidin B2 in mitigating NAFLD with oxidative stress. To gain a deeper understanding of the component’s effects on NAFLD, we utilized subsequent experiments to evaluate the effects of procyanidin B2. We found that procyanidin B2 inhibited lipogenesis and modulated related signaling pathways in vivo. These results showed that procyanidin B2 suppressed the T090-induced expression of SREBP-1c in a concentration-dependent manner (Figure 5B). We also found that procyanidin B2 increased the phosphorylation of AMPK and YAP (Figure 5C) and enhanced the expression of HO-1 (Figure 5D). In summary, our findings suggest that procyanidin B2, a potential quality marker for SC, played a crucial role in mitigating NAFLD with oxidative stress by modulating key proteins and signaling pathways, such as AMPK and YAP.

## 4. Discussion

Elevated oxidative stress plays a significant role in liver pathogenesis, contributing to various stages such as cellular damage, inflammation, fibrosis, and cirrhosis. While mitigating oxidative stress may be advantageous for NAFLD management, pinpointing potential candidates and understanding their underlying mechanisms remain challenging tasks. In this study, we successfully investigated the antioxidant properties of SC and its impact on NAFLD using a multiscale network-level approach combined with experimental validation. The integrated approach allowed us to elucidate the hepatoprotective effects of SC, as well as the key mechanisms and active ingredients underlying these effects. Our findings provided the identification of key targets and significantly associated the signaling pathways of SC, which were further validated through in vitro and in vivo experiments. Additionally, we identified the active ingredient procyanidin B2 as a potential therapeutic agent for NAFLD. We believe that these findings provided valuable insights into the potential applications of herbal medicines, such as SC, and in the development of more effective therapeutic strategies for NAFLD.

Unlike previous studies, our study presents an integrated framework that combines network pharmacology analysis and experimental validation, making an important contribution to the exploration of system-level mechanisms in herbal medicine. Prior investigations have often relied on protein overlap or protein–protein interactions (PPIs) between drug targets and disease proteins to understand therapeutic effects. Our study, however, expanded on this approach by incorporating not only PPIs but also the relationships between biological functions, enabling a more comprehensive elucidation of key components and their underlying mechanisms. In addition, some early studies only presented hypotheses based on network pharmacology analysis without experimental validation. In our research, we comprehensively performed experimental validation for both the therapeutic effects and the identified active ingredients, enhancing the reliability of our findings. Collectively, our results introduce a novel methodology for a more comprehensive understanding of the mechanisms of herbal medicine, which hold the potential to make a significant impact in the field.

The results of the network pharmacological analysis investigated the complex relationships among the compounds, targets, and biological effects of SC on NAFLD. This comprehensive analysis provided insights into the potential interactions between SC ingredients and their protein targets concerning NAFLD. By constructing a compound-target network of SC using various databases, we initially identified 589 CPIs involving 21 SC ingredients and 321 protein targets, with related proteins associated with oxidative stress. Focusing on 111 targets that interacted with two or more ingredients, our subsequent analysis revealed a significant 22-fold overlap between the key targets of SC and oxidative stress (*p*-value < 10^−30^), emphasizing the potential of SC as a therapeutic agent for NAFLD. Through an over-representation analysis based on the KEGG database, we discovered that the majority of the signaling pathways were significantly associated with the main targets of SC. These findings have guided in vitro and in vivo experimental trials while also highlighting the complex and multifaceted nature of the potential for treating NAFLD. Although our research showcased integrated approaches, we primarily focused on an HFD model to evaluate the efficacy of SC via oral administration. Future interesting studies may involve investigating the effects of SC on various models, such as different species, including rats, with a HF diet or genetically fatty liver. Utilizing these different models to explore the capacity of SC may lead to a better understanding of the therapeutic potential of SC in different contexts and conditions.

Our results confirm that the protective effects of SC against NAFLD are mainly mediated by the AMPK and YAP signaling pathways. AMPK, a crucial sensor of cellular energy levels, is activated in response to various conditions that have a falling cellular energy status, including nutrient deprivation, such as glucose, hypoxia, physical exercise, and exposure to toxicants [35]. AMPK, which is activated by LKB1, is an upstream kinase that is responsive to increases in the ratios of AMP:ATP and ADP:ATP and activates catabolic pathways, including glycolysis, glucose uptake via GLUT4 and GLUT1, fatty acid oxidation, fatty acid uptake, mitochondrial biogenesis, and autophagy. Meanwhile, it inhibits anabolic pathways, including triglyceride synthesis, fatty acid synthesis, cholesterol synthesis, glycogen synthesis, protein synthesis, and rRNA synthesis [36]. The activation of AMPK has been considered a potential therapeutic strategy for improving NAFLD, as numerous preclinical animal models have shown its efficacy, and it is known that AMPK activity decreases in many conditions associated with NAFLD, such as inflammation, obesity, and diabetes [37]. In HFD-induced NAFLD mouse liver tissues, the activation of AMPK and Nrf2 by Liensinine inhibited NOX4 and consequently restrained ROS production and inhibited lipid accumulation [38]. Our previous research has confirmed that SC activates AMPK in liver cells [13]. Based on this finding, we anticipate that SC may have the potential to improve NAFLD models through the activation of AMPK.

The Hippo–YAP pathway is a signaling pathway that regulates organ size and growth and plays essential roles in cell proliferation, differentiation, and apoptosis [39]. Yap has been proposed as an oncogenic candidate, while other components of the Hippo pathway function have been proposed as tumor suppressors. The dysregulation of the Hippo pathway has been associated with the disruption of contact inhibition and tissue growth control in cancer cells [40]. The activation of YAP has been implicated in cancer initiation, progression, and metastasis, as it promotes abnormal cell proliferation, invasion, and tissue growth control in cancer cells [41]. YAP can also promote the expression of genes related to lipogenesis by interacting with SREBPs in FAS and HMGCR promoters. UCP1 transcription in the brown adipose tissue requires YAP, uncoupling oxidative phosphorylation [42]. The YAP-induced upregulation of serum- and glucocorticoid-regulated kinase 1 (SGK1) activates mTORC1 and SREBP, leading to the stimulation of de novo lipogenesis [43]. AMPK activation is involved in the phosphorylation of LATS and direct phosphorylation of YAP’s Ser94 residue, resulting in a disruption in the interaction between YAP and TEAD [44]. This indicates a link between AMPK and YAP, and our research further confirmed the involvement of the AMPK-YAP pathway in the regulation of lipogenesis and alleviation of oxidative stress caused by lipogenesis. The results of our comprehensive experiments suggest that procyanidin B2 may be the active ingredient responsible for the effects of SC. Procyanidin B2, contained in grape seeds, cacao beans, and apples [45], has been identified as a bioactive component of SC. Procyanidin B2 has been reported to exhibit protective effects against acute lung injury, cardiomyocyte fibrosis, and blood–brain barrier disruption [46,47,48]. It has been reported that Procyanidin B2 inhibits colorectal cancer cells through the induction of autophagy via the regulation of a PI3K/Akt signaling pathway [49] and protects against acute kidney injury by improving mitochondrial dynamics through Nrf2 nuclear translocation [50]. Additionally, Procyanidin B2 has been shown to protect podocytes by inhibiting mitochondrial dysfunction and apoptosis through the activation of the AMPK-SIRT1-PGC-1α signaling pathway [51]. In particular, Procyanidin B2 was revealed to have improved diet-induced obesity, NAFLD, and dyslipidemia [52,53,54]. Therefore, we can consider Procyanidin B2 as a promising compound with potential as an antioxidant and lipogenesis inhibitor.

## 5. Conclusions

Our study proposed an integrated approach to uncover the antioxidant properties of SC on NAFFLD. We identified its putative mechanism and the key components of SC through analysis on a multiscale network. In vivo, we confirmed that SC alleviated HFD-induced hepatic fat accumulation, diabetes, hyperlipidemia, and liver tissue damage. In vitro, we validated the regulation of the key signaling pathway, the AMPK-YAP pathway, and identified Procyanidin B2 to be the major active compound. These results suggest that SC and its major compound, Procyanidin B2, have potential as antioxidants and lipogenesis inhibitors.

## Figures and Tables

**Table 1:** Significant enrichment pathway related to oxidative stress and NAFLD by key targets of *Spatholobi caulis* (adjusted *p*-value of ≤0.05).

Term	Overlap	*p*-Value (Adjusted *)	Combined Score	Genes
AMPK signaling pathway	8/120	3.15 × 10^−6^	173.74	*CCND1*; *FASN*; *AKT1*; *PPARG*; *PIK3CB*; *HMGCR*; *SIRT1*; *IGF1R*
Apoptosis	19/142	2.60 × 10^−20^	1708.92	*JUN*; *S TAT1*; *PIK3CB*; *FOS*; *MAPK14*; *TNF*; *NFKB1*; *RELA*; *NFKBIA*; *IL6*; *MAPK8*; *IL1B*; *AKT1*; *MAPK1*; *TLR4*; *MAPK3*
JAK-STAT signaling pathway	12/162	9.40 × 10^−10^	331.54	*IL4*; *IL6*; *CDKN1A*; *CCND1*; *THPO*; *STAT1*; *STAT3*; *BCL2*; *AKT1*; *PIK3CB*; *EGFR*; *IL2*
MAPK signaling pathway	19/294	2.62 × 10^−14^	482.94	*CDKN1A*; *BAD*; *NOS3*; *PTEN*; *PRKCA*; *PIK3CB*; *RELA*; *EGFR*; *NFKB1*; *IL2*; *IGF1R*; *VEGFA*; *COL1A1*; *IL4*; *CASP9*; *IL6*; *CCND1*; *BCL2*; *AKT1*; *MAPK1*; *TP53*; *TLR4*; *MAPK3*
NF-kappa B signaling pathway	13/104	1.66 × 10^−13^	433.42	*JUN*; *PRKCB*; *PRKCA*; *FOS*; *MAPK14*; *TNF*; *RELA*; *EGFR*; *NFKB1*; *IGF1R*; *VEGFA*; *MAPK8*; *IL1B*; *CASP3*; *AKT1*; *MAPK1*; *MAPT*; *TP53*; *MAPK3*
PI3K-Akt signaling pathway	23/354	1.99 × 10^−17^	1109.84	*VCAM1*; *PARP1*; *PRKCB*; *PTGS2*; *TNF*; *NFKB1*; *RELA*; *ICAM1*; *NFKBIA*; *LCK*; *IL1B*; *BCL2*; *TLR4*
Toll-like receptor signaling pathway	16/104	4.21 × 10^−18^	1327.81	*JUN*; *PARP1*; *BAD*; *PIK3CB*; *FOS*; *TNF*; *RELA*; *NFKB1*; *NFKBIA*; *CASP9*; *MAPK8*; *CASP3*; *LMNA*; *BCL2*; *BAX*; *AKT1*; *MAPK1*; *TP53*; *MAPK3*

* *p*-values were adjusted for multiple testing using the Bonferroni correction method.

**Table 2:** Representative ingredients of *Spatholobi caulis* and its association with NAFLD and oxidative stress.

Name	PubChem ID	Structure	Overlap (*p*-Value ^#^)	Correlation Score *
(−)-Epicatechin	72276	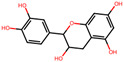	8/18 (8.74 × 10^−13^)	0.094
Genistein	5280961	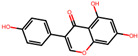	19/64 (1.04 × 10^−23^)	0.085
Procyanidin B2	122738	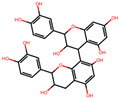	3/7 (6.21 × 10^−6^)	0.085
Formononetin	5280378	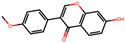	7/19 (1.35 × 10^−10^)	0.084
Daidzein	5281708	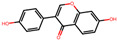	15/53 (1.39 × 10^−18^)	0.079
Hesperetin	72281	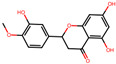	8/25 (3.45 × 10^−11^)	0.075
Butein	5281222	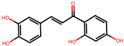	7/23 (8.44 × 10^−10^)	0.070
Liquiritigenin	114829	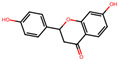	3/10 (3.63 × 10^−5^)	0.070
Prunetin	5281804	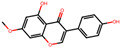	1/6 (1.70 × 10^−3^)	0.068
Isoliquiritigenin	638278	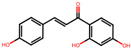	8/32 (2.59 × 10^−11^)	0.063

^#^ *p*-values are computed by hypergeometric test. * Pearson’s correlation between diffusion profile of CS ingredients and disease calculated in multiscale network.

## Data Availability

All of the data is contained within the article.
